# Azygos vein preservation is feasible and beneficial in esophageal atresia with tracheoesophageal fistula: A meta-analysis of randomized controlled trials

**DOI:** 10.3389/fped.2022.965275

**Published:** 2022-07-28

**Authors:** Chuan Wang, Junkai Zheng, Xue Ma

**Affiliations:** Department of Pediatric Surgery, West China Hospital of Sichuan University, Chengdu, China

**Keywords:** esophageal atresia, azygos vein, complication, anatomic leak, mortality

## Abstract

**Background:**

Esophageal atresia (EA) with tracheoesophageal fistula (TEF) is a common congenital anomaly. It is still unknown whether azygos vein preservation will increase the difficulty or time of operation and reduce the quality of anastomosis. Thus, we conducted this meta-analysis to explore the puzzle.

**Methods:**

Two researchers independently searched the databases. Randomized controlled trials were included if these studies applied thoracotomy to perform operations and compared the outcomes in patients with EA/TEF between azygos vein preservation groups and azygos vein ligation groups. The Jadad score was used to assess the quality of the included studies. Statistical heterogeneity was evaluated using the *I*^2^ value. A fixed or random-effect model was applied regarding the *I*^2^ value.

**Results:**

Four studies involving 286 patients were included. The pooled estimates indicated that preservation of the azygos vein decreased the incidence of anatomic leakage with a pooled risk ratio (RR) of 0.54 (95% CI 0.29–0.99, *P* = 0.05) and mortality with an RR of 0.51 (95% CI 0.29–0.90; *P* = 0.02). Preservation of the azygos vein might not require a longer operative time than ligation of the azygos vein.

**Conclusions:**

This research certifies that preservation of the azygos vein is able to reduce the prevalence of anastomotic leakage and mortality.

## Introduction

Esophageal atresia (EA) is a common congenital anomaly of the digestive tract, with a prevalence of 1 in 3,500 births (1). EA with tracheoesophageal fistula (EA/TEF) accounts for ~70–90% of cases of EA (2, 3). Operation is indispensable to save the patient's life. The azygos vein is usually ligated and divided to facilitate operation during the repair procedure of EA/TEF (4).

The azygos vein is an important vein that ascends in the posterior region of the right thorax and next to the vertebral column. It drains blood from all of the posterior intercostal veins but the first intercostal vein in the right thorax into the superior vena cava. Some investigators maintain that the azygos vein is a major vessel and should be preserved if preservation does not augment the incidence of postoperative complications (5). Anastomotic leakage is one of the most frequent postoperative complications and significantly increases postoperative hospitalization time and cost (6, 7). Some researchers believe that ligation of the azygos vein will aggravate postoperative edema at the anastomosis and chest congestion and consequently lead to higher incidences of complications, including anastomotic leakage and pneumonitis (5, 8). Although a series of studies have been performed to verify this assumption, those studies have failed to achieve consistent conclusions (5, 8–11). Concerns are raised regarding whether azygos vein preservation will increase the difficulty or time of operation and reduce the quality of anastomosis. A longer operative time results in longer anesthetic exposure. Longer anesthetic exposure may augment the risk of impairment in neural development (12). With the view that more than 90% of pediatric surgeons preferring thoracotomy to thoracoscopy to deal with EA, (13) in the present study, we conducted this meta-analysis to investigate whether azygos vein preservation would be feasible with no increased risks or even reduced risks of postoperative complications in patients undergoing thoracotomy.

## Methods

### Study selection

This review was reported in accordance with the Preferred Reporting Items for Systematic Reviews and Meta-analyses statement (14). Randomized controlled trials (RCTs) were included if these studies applied thoracotomy to perform operations and compared the outcomes in patients with EA/TEF between azygos vein preservation groups and azygos vein ligation groups. Patients in the included studies should receive primary repair procedures rather than staged procedures. Meanwhile, eligible studies were mandatory to report at least one of the predetermined outcomes, including operative time, anastomotic leak and mortality. The included studies were restricted to studies published in English.

### Search strategy

The search strategy was designed and conducted by two researchers (C.W. and J.W.). We independently searched databases including PubMed, EMBASE and the Cochrane Library. The keywords containing “azygos vein” and “esophageal atresia” were combined with the Boolean operator AND to identify published potential studies. Each of the two researchers independently reviewed the titles and abstracts to refine the results. Full-text manuscripts of the relevant studies were scrutinized to identify potential studies that met the inclusion criteria. Reference lists of included studies were inspected to screen additional studies.

### Data extraction and quality assessment

Anastomotic leakage was defined as the primary outcome. Operative time and mortality were defined as the secondary outcomes. Two authors (C.W. and J.W.) independently abstracted and documented the following information from eligible studies: lead author, publication year, sample size, study design and outcomes. The Jadad score was adopted to assess the quality of the included studies. The Jadad score, ranging from 0 to 5, was applied to evaluate the quality of RCTs, and a study with a score >2 was regarded as a “high quality” study (15).

### Statistical analysis and exploration of heterogeneity

Reviewer Manager 5.3 from the Cochrane Collaboration was applied to perform the meta-analysis. We adopted the Mantel–Haenszel statistical method to calculate risk ratios (RRs) and 95% confidence intervals (CIs) for the pooled results of all outcomes in the meta-analysis. The potential bias for publication was evaluated by employing funnel-plot symmetry. Statistical heterogeneity among summary data was assessed by the *I*^2^ method, with an *I*^2^ value higher than 50%, suggesting substantial heterogeneity. If the *I*^2^ value was higher than 50%, a random-effects model of analysis was applied; if not, a fixed-effects model was applied.

## Results

**Figure 1:**
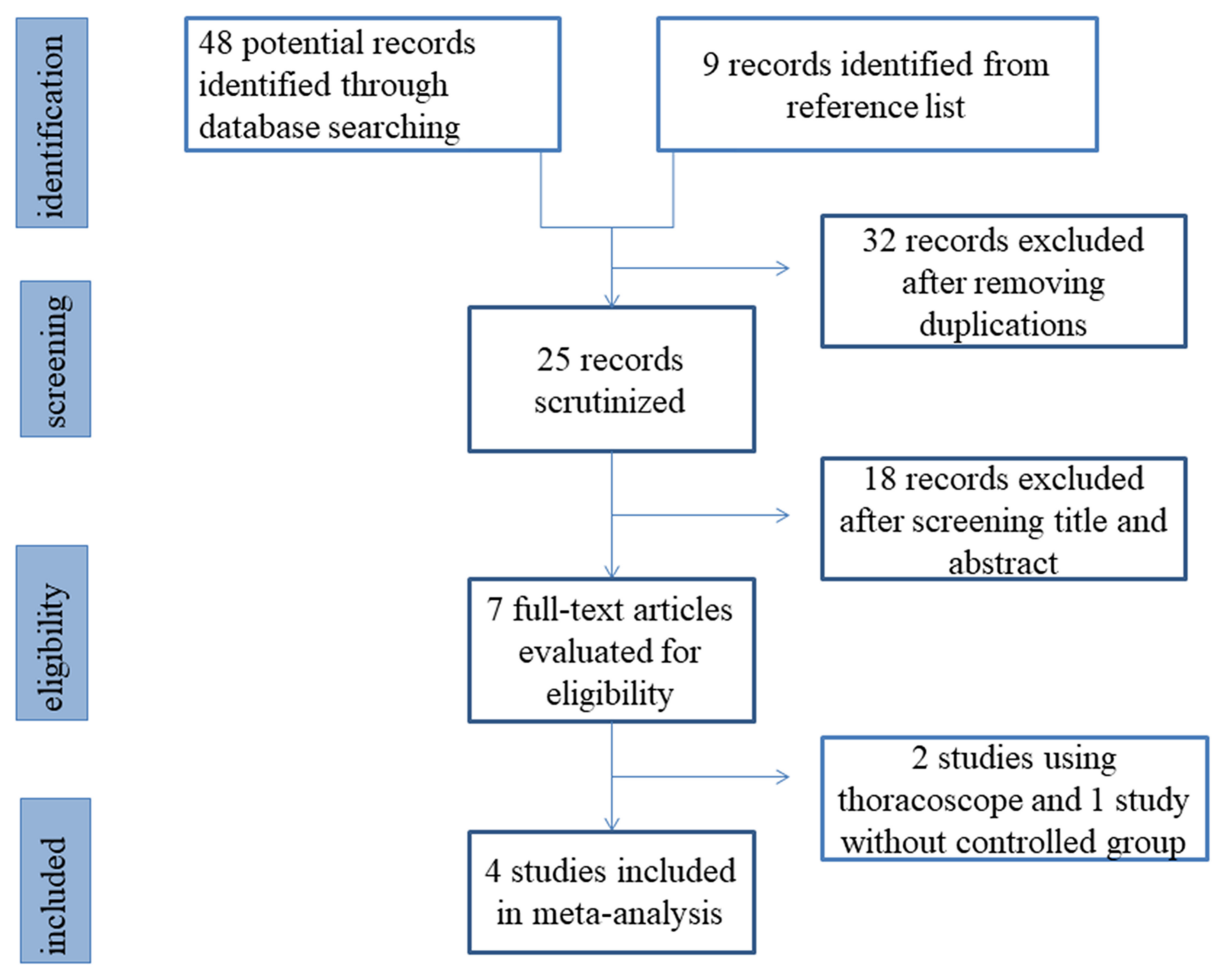
Flow chart of study selection.

Study selection and the results of selection are shown in Figure 1. Forty-eight potentially eligible records were identified by using the search strategy. After carefully reviewing the titles and abstracts of these publications, the full texts and reference lists of 7 records were scrutinized, and 4 articles meeting the inclusion criteria were ultimately included in the final analyses (5, 8, 10, 11). Table 1 summarizes the baseline characteristics and Jadad scores of these four studies. A total of 286 patients were assigned to the preservation group (*n* = 164) or the ligation group (*n* = 122). Table 2 reports the exact case numbers of each outcome in each study. There was no evidence of obvious publication bias in each analysis, based on funnel-plot symmetry.

**Table 1:** Characteristics of included studies.

**Study**	**Study type**	**Surgery approach**	**Sample size**	**Mean age (h)**	**Sex**	**Weight (kg)**	**Associated anomalies**	**Gap length (cm)**	**JS**
Upadhyaya et al. (8)	RCT	Thoracotomy	Preservation: 25	NA	Male/Female:NA	NA	8	NA	3
			Ligation: 25	NA	Male/Female:NA	NA	9	NA	
Sharma et al. (5)	RCT	Thoracotomy	Preservation: 46	NA	Male/Female:33/13	2.32	12	NA	8
			Ligation: 50	NA	Male/Female:27/23	2.52	14	NA	
Rashid et al. (11)	RCT	Thoracotomy	Preservation: 81	41.07	Male/Female:49/32	2.66	24	1.33	2
			Ligation: 35	37.93	Male/Female:22/13	2.48	13	1.43	
Fathi et al. (10)	RCT	Thoracotomy	Preservation: 12	36.25	Male/Female:7/5	2.36	NA	NA	8
			Ligation: 12	34.66	Male/Female:6/6	2.22	NA	NA	

*RCT, randomized controlled trial; NA, not available; JS, Jadad score*.

**Table 2:** Summary of the outcomes of included studies.

**Study**	**Sample size**	**Anastomotic leakage**	**Mortality**	**Mean operative time (min)**
Upadhyaya et al. (8)	Preservation: 25	4 (16.00%)	4 (16.00%)	80
	Ligation: 25	5 (20.00%)	4 (16.00%)	70
Sharma et al. (5)	Preservation: 46	3 (6.52%)	5 (10.87%)	60
	Ligation: 50	10 (20.00%)	14 (28.00%)	64
Rashid et al. (11)	Preservation: 81	10 (12.35%)	9 (11.11%)	63
	Ligation: 35	7 (20.00%)	8 (22.85%)	60
Fathi et al. (10)	Preservation: 12	0 (0.00%)	NA	60
	Ligation: 12	0 (0.00%)	NA	60

*NA, not available*.

### Operative time

Although all four records reported the average operative time (5, 8, 10, 11), only one record reported the average operative time with standard deviation (10). The results of three studies found a longer mean operative time in the preservation group than in the ligation group, (8, 10, 11) ranging from 0 to 10 min, while one study showed a longer mean operative time in the ligation group than in the preservation group (5).

### Anastomotic leakage

**Figure 2:**
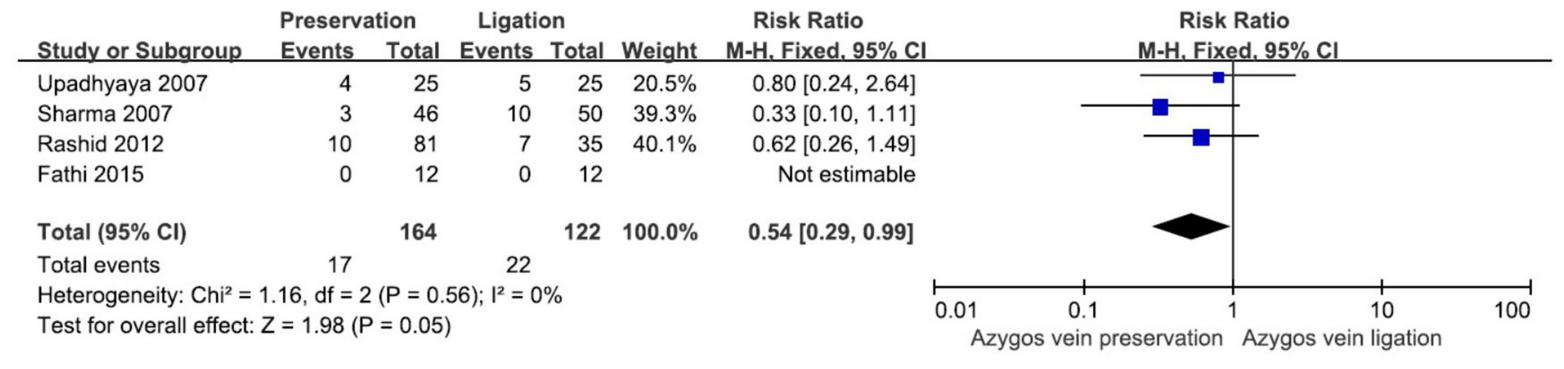
Forest plot showing the risk ratio for the occurrence of anastomotic leakage in the two groups.

All four studies reported the incidence of anastomotic leakage as an outcome in pediatric patients with EA/TEF (5, 8, 10, 11). The incidence of anastomotic leakage was 10.37% (17, *n* = 164) in the preservation group and 18.03% (22, *n* = 122) in the ligation group. No detectable heterogeneity was examined among the four studies (*I*^2^= 0%). The results of the meta-analysis indicated that there was a perceptible discrepancy in the incidence of anatomic leakage between the two groups, with a pooled RR of 0.54 (95% CI 0.29–0.99, *P* = 0.05) (Figure 2). The preservation group had a lower risk of anastomotic leakage than the ligation group.

### Mortality

**Figure 3:**
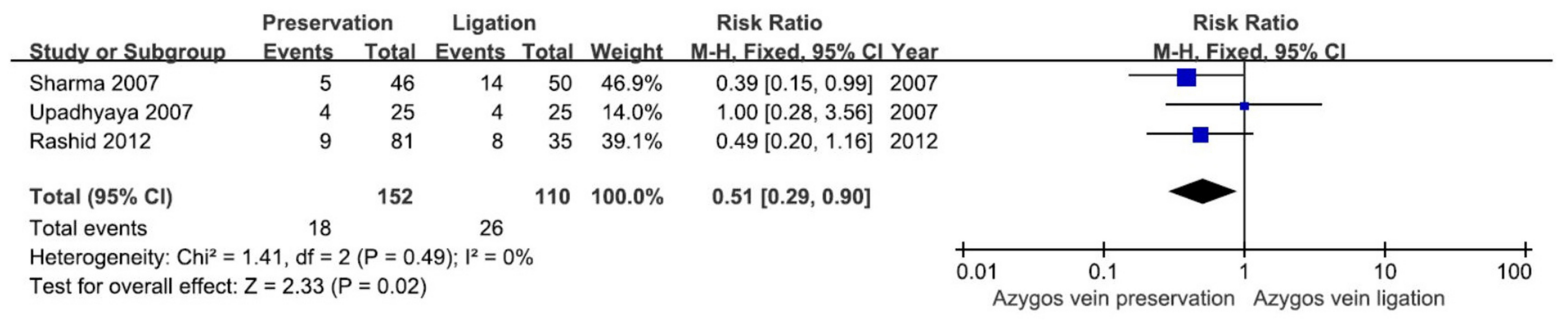
Forest plot showing the risk ratio for the occurrence of mortality in two groups.

Three out of four studies investigated mortality in EA/TEF patients (5, 8, 11). In total, there were 18 deaths in the preservation group (*n* = 152) and 26 deaths in the ligation group (*n* = 110). There was no observable heterogeneity among these studies (*I*^2^= 0%). A significant difference in mortality was detected between the two groups, and the ligation group had a higher risk of mortality (RR 0.51, 95% CI 0.29–0.90; *P* = 0.02) (Figure 3).

## Discussion

EA is the most frequent congenital malformation of the esophagus, which is supposed to emerge as a consequence of the abnormal development of the foregut (16). The first successful end-to-end anastomosis and fistula ligation to treat EA/TEF was presented in 1941, (17) and currently, surgery is considered to be inevitable to save life.

In the standard operation procedure of repair surgery of EA/TEF, the azygos vein would be ligated and divided to provide a clear operation field and facilitate identification of the distal esophagus and fistula to conduct esophageal anastomosis. It was worrisome whether preservation of the azygos vein would prolong operative time. A longer duration of surgery means a longer duration of general anesthesia. Some studies have indicated that prolonged anesthesia might affect neurodevelopmental outcomes (18). Harmsen et al. reported that the duration of anesthetic exposure was negatively correlated with motor developmental outcome in patients with EA (19). Our study found that the preservation group might not need a longer operative time than the ligation group. The results of four studies were not consistent. The differences in the mean operative times in the two groups were small. Further large trials are needed to clarify this question.

The azygos vein drains deoxygenated blood from the ascending lumbar veins and the right subcostal veins into the superior vena cava, which means that the azygos vein also drains the esophagus and bronchus. Compared with ligation of the azygos vein, preservation of the azygos vein possibly lowered the degree of esophageal anastomotic edema and postoperative congestion of the lung. It might be reasonably deduced that preservation of the azygos vein could reduce the risks of anastomotic complications and pneumonias.

Anastomotic leak was a main early postoperative complication in EA patients, with an estimated prevalence of 20% (20). Patients with long gap EA had a higher risk of anastomotic leakage, which might be attributed to high tension on the anastomosis (20, 21). Minor leaks might heal spontaneously by managing conservatively with drainage. Nevertheless, major leakage might be life-threatening, and reoperation may be needed. Some researchers supposed that ligation of the azygos vein would aggravate postoperative edema at the anastomosis and consequently increase the risk of anastomotic leakage in patients (8). Our study demonstrated that preservation of the azygos vein could reduce the prevalence of anastomotic leakage. Nevertheless, whether the decrease in anastomotic leakage is related to the relief of postoperative edema by draining blood remains to be clarified.

Although mortality after EA repair remarkably dropped from 81% in the 1940s to 9% in the 2010s with the development of equipment and perioperative management 26, death is still a threat to patients with EA. In this study, we found that preservation of the azygos vein could significantly lower mortality. Approximately half of patients with EA have associated anomalies (3, 22). Cardiac defects were the most common associated anomaly, with a prevalence of 26.7% (22). It was reported that cardiac defects were related to mortality (22, 23). Preservation of the azygos vein might have less of an effect on the circulatory system than ligation of the azygos vein. In addition, preservation of the azygos vein was able to reduce the risk of postoperative pneumonitis (8, 11). These advantages might contribute to the lower mortality.

Some limitations should be noted in this study. Although only RCTs were included in this meta-analysis, which maximally reduced potential biases, the number of included studies was limited. Many postoperative complications, including anastomotic stricture, recurrent TEF and gastroesophageal reflux disease, were not compared owing to a lack of relevant data. Thus, we were not able to evaluate the preservation of azygos veins in all aspects, and more studies are needed to assess the preservation of azygos veins in those aspects to provide a further understanding.

## Conclusions

This study offers distinguished evidence regarding the safety and benefit of preservation of the azygos vein in patients with EA/TEF. Our research demonstrates that preservation of the azygos vein is able to reduce the prevalence of anastomotic leakage and mortality compared with ligation of the azygos vein. Further studies are needed to evaluate the preservation of the azygos vein in other aspects, including anastomotic stricture, recurrent TEF and gastroesophageal reflux disease.

## Data availability statement

The original contributions presented in the study are included in the article/supplementary material, further inquiries can be directed to the corresponding author.

## Author contributions

XM contributed to the design of the study. CW and JZ performed the literature search and extracted the data. XM and CW analyzed the data and interpreted the statistical analysis. XM, CW, and JZ drafted the manuscript. All authors read and approved the final manuscript.

## Conflict of interest

The authors declare that the research was conducted in the absence of any commercial or financial relationships that could be construed as a potential conflict of interest.

## Publisher's note

All claims expressed in this article are solely those of the authors and do not necessarily represent those of their affiliated organizations, or those of the publisher, the editors and the reviewers. Any product that may be evaluated in this article, or claim that may be made by its manufacturer, is not guaranteed or endorsed by the publisher.

## Abbreviations

CI: Confidence interval
EA: Esophageal atresia
TEF: Tracheoesophageal fistula
RCT: Randomized controlled trial
RR: Risk ratio.
